# Carbon Dioxide Reactivity of Brain Tissue Oxygenation after Pediatric Traumatic Brain Injury

**DOI:** 10.3390/children9030409

**Published:** 2022-03-14

**Authors:** Damla Hanalioglu, Ann Oh, M’Hamed Temkit, P. David Adelson, Brian Appavu

**Affiliations:** 1 Barrow Neurological Institute at Phoenix Children’s Hospital, Phoenix, AZ 85016, USA; dhanoglu@gmail.com (D.H.); mtemkit@phoenixchildrens.com (M.T.); dadelson@phoenixchildrens.com (P.D.A.); 2 Department of Neurology, University of Colorado School of Medicine, Aurora, CO 80045, USA; ann.oh@cuanschutz.edu

**Keywords:** traumatic brain injury, brain tissue oxygenation, carbon dioxide reactivity

## Abstract

Background: We investigated how changes in partial pressure of brain tissue oxygenation (PbtO_2_) relate to end-tidal carbon dioxide (EtCO_2_) after pediatric traumatic brain injury (TBI). Methods: Dynamic structural equation modeling (DSEM) was used to investigate associations between EtCO_2_ and PbtO_2_, with positive associations indicating intact CO_2_ reactivity of PbtO_2,_ and negative associations indicating impaired reactivity. Sub-analyses were performed to investigate associations of PbtO_2_ to intracranial pressure (ICP), arterial blood pressure (ABP) and cerebral regional oximetry (rSO_2_). Results: Among 14 patients, a positive association between PbtO_2_ and EtCO_2_ was demonstrated (SRC 0.05, 95% CI [0.04, 0.06]), with 9 patients demonstrating intact CO_2_ reactivity and 5 patients demonstrating impaired reactivity. Patients demonstrating intact CO_2_ reactivity had positive associations between PbtO_2_ and ICP (0.22 [0.21, 0.23]), whereas patients with impaired reactivity had negative associations (−0.28 [−0.29, −0.28]). Patients demonstrating intact CO_2_ reactivity had negative associations between PbtO_2_ and rSO_2_ (−0.08 [−0.09, −0.08]), whereas patients with impaired reactivity had positive associations (−0.15 [0.14, 0.16]). Compared to patients with intact CO_2_ reactivity_,_ those with impaired reactivity had increased ICP (*p* < 0.0000), lower PbtO_2_ (*p* < 0.0000) and higher PRx (*p* = 0.0134). Conclusion: After TBI, CO_2_ reactivity of PbtO_2_ can be heterogenous, necessitating further work investigating factors contributing toward impaired reactivity.

## 1. Introduction

Traumatic brain injury (TBI) is the most common cause of death and disability among children and young adults worldwide [1,2]. Current management of TBI is based on Brain Trauma Foundation (BTF) guidelines [3]; however, these include only Level II and III recommendations. Despite numerous clinical trials, high quality evidence is still absent to guide clinical management of TBI toward optimization of long-term functional outcomes [4]. Existing management strategies focus on the prevention of secondary brain insults that may contribute toward worsened outcomes, including but not limited to maintenance of adequate cerebral blood flow (CBF) and oxygenation.

In order to mitigate secondary brain injury and improve long-term outcomes, optimization of brain tissue oxygenation is an important element to pediatric TBI management. Continuous measurements of the partial pressure of brain tissue oxygenation (PbtO_2_) can be monitored using a micro-Clark electrode surgically implanted within brain parenchyma [5]. Level III recommendations exist in current TBI guidelines to maintain PbtO_2_ values > 10 mmHg [3]. The mechanisms to optimize brain tissue oxygenation and avoid hypoxia include maintenance of appropriate cerebral perfusion and adjustment of ventilation to influence arterial content of partial pressure of carbon dioxide (PaCO_2_) [6]. Carbon dioxide is a potent vasodilator of cerebral small vessel arterioles, with changes in PaCO_2_ directionally related to alterations in CBF [7]. Cerebrovascular carbon dioxide (CO_2_) reactivity is a relatively linear response within physiological ranges. However, beyond the limits of vasoconstrictive and vasodilatory capacity, alterations in PaCO_2_ may induce derangements in CBF [7]. Since carbon dioxide (CO_2_) also affects cerebrovascular resistance, deliberate changes in CO_2_ partial pressure have been considered as useful to manipulate CBF regulation in an environment of impaired autoregulation and disrupted blood brain barrier (BBB) [8]. However, limited information is available regarding temporal relations of PbtO_2_ and PaCO_2_ after severe TBI. In a few studies, researchers reported that hyperventilation to induce cerebral vasoconstriction and reduce CBF, intracranial pressure (ICP), and cerebral blood volume may unintentionally lead to brain tissue hypoxia after TBI. Furthermore, adjustments in PaCO_2_ to optimize PbtO_2_ may not be effective if CO_2_ vasoreactivity is diminished [7]. The magnitude and extent of BBB breakdown carries potential critical implications regarding what neuroprotective measures in neurocritical care may optimize or even deteriorate brain oxygenation and homeostasis [9].

In this study, we aimed to identify times series associations between end-tidal carbon dioxide content (EtCO_2_) and PbtO_2_ as well as their relationships with intracranial pressure (ICP), arterial blood pressure (ABP) and cerebral regional oxygen saturation (rSO_2_). We hypothesized that in the context of time series, and EtCO_2_ and PbtO_2_ are associated with each other, but the direction of the association may change depending on the status of the systemic and local tissue characteristics of the injured brain.

## 2. Materials and Methods

### 2.1. Study Design

This is a retrospective study from a prospective clinical database. The study was conducted at Phoenix Children’s Hospital (PCH) and was approved by the PCH Institutional Review Board (IRB: 20-284).

Pediatric patients (<21 years of age) with TBI from a single pediatric intensive monitoring unit were retrospectively analyzed, undergoing multimodality neurologic monitoring that included continuous synchronized measurements of ICP, ABP, EtCO_2_, rSO_2_ and PbtO_2_. ABP monitoring was assessed from a radial arterial line. ICP monitoring was performed using an intraparenchymal probe (Codman ICP Monitor, Integra Life Sciences, Billerica, MA, USA). EtCO_2_ was monitored by capnograph connected to the endotracheal tube. PbtO_2_ monitoring was performed using an intraparenchymal micro-Clark electrode (Integra Life Sciences, Billerica MA, USA). rSO_2_ monitoring was monitored using the Covidien INVOS System (Medtronic, Minneapolis, MN, USA). Patients were managed according to institutional guidelines founded upon the most up to date pediatric TBI guidelines at the time [3,10]. 

### 2.2. Patients

Demographic patient information included age, sex, and race. Injury characteristics included Glasgow Coma Scale (GCS) score at presentation on day of admission. GCS scores range from 3 to 15 with lower scores indicative of higher injury severity. Primary injury mechanisms were also described including closed, crush, and penetrating injuries [11]. Functional outcome characteristics included Glasgow Outcome Scale—Extended Pediatrics (GOSE-Peds) collected at 12 months post-injury [12]. GOSE-Peds scores range from 1 to 8 with higher scores indicative of worsened outcomes.

### 2.3. Physiologic Data

Patients underwent multimodality neurologic monitoring (MMM), which included integration of ICP, ABP, EtCO_2_, rSO_2_, and PbtO_2_ monitoring. Patients underwent rSO_2_ monitoring either with a single probe on the forehead or bilateral probes on each hemisphere. When bilateral rSO_2_ monitoring was performed, the monitoring data ipsilateral to the PbtO_2_ probe was assessed. Continuous physiologic data from all of the monitoring devices were collected and time-synchronized using an MMM device (Moberg CNS200; Moberg ICU Solutions, Philadelphia, PA, USA). ICM+ software (Cambridge, UK) was used to visualize data and export synchronized time series physiologic data at 1 Hz. Data was collected in 5-h epochs on the first day of recording and after PbtO_2_ calibration was complete. In addition to the above-described physiologic data, we also collected the pressure reactivity index (PRx). PRx is a moving Pearson correlation coefficient relating ICP and ABP with a calculation period of 300 s updated every 60 s. PRx is an indicator of cerebrovascular pressure reactivity (CVPR) with values approaching −1 representing efficient CVPR and vales approaching 1 representing inefficient CVPR [13,14,15]. We implemented artifact reduction by excluding timepoints in which there were missing values for any physiologic variable, as well as epochs in which physiologic values were sub-physiologic or supraphysiologic of acceptable ranges. This included utilizing normative ranges of ICP between >0 and 90 mmHg, EtCO_2_ between 0 and 60 mmHg, ABP between 25 and 140 mmHg, rSO_2_ between 5–99%, and PbtO_2_ between >0 and 90 mmHg. Serum hemoglobin levels were drawn during the analysis period or just before it was collected. ABP, ICP, EtCO_2_, rSO_2_, PbtO_2_, PRx and hemoglobin data were summarized using descriptive statistics including the median value and interquartile range [IQR].

### 2.4. Pharmacologic Data

To explore whether pharmacological agents may play a role in physiologic patterns, we described the use of sedative, vasoactive, and hyperosmolar agents used during the analysis period for each patient. Sedative pharmacotherapy included fentanyl, morphine, propofol and dexmedetomidine. Vasoactive agents included norepinephrine and epinephrine. Hyperosmolar agents included 3% hypertonic saline.

### 2.5. Statistical Analyses

Demographic data was summarized using descriptive statistics including median and IQR. The strength of association of PbtO_2_ to ABP, ICP, rSO_2_ and EtCO_2_ was investigated using multivariate dynamic structural equation modeling (DSEM) both at the subject level as well as through grouped and sub-grouped analyses. DSEM is estimated with Bayesian inference methods using the Markov chain Monte Carlo Gibbs sampler and the Metropolis-Hasting sample and is used for analyzing intensive longitudinal data where observations from single or multiple subjects are collected at many points in time [16]. Statistical significance for associations was determined by ascertaining that the 95% credible interval (95% CI) for the standardized regression coefficient (SRC) from DSEM models did not include 0. Significant differences in strength of associations were determined by observing absence of overlap in 95% CI between the groups investigated. The value of SRC reflects the strength of linear associations between the studied physiologic variables. Given known properties of chemoregulation of CBF and its association with cerebral oxygenation, intact CO_2_ reactivity of PbtO_2_ was characterized as having a significant positive relationship with EtCO_2_. Likewise, impaired CO_2_ reactivity of PbtO_2_ was characterized as having a significant negative relationship between PbtO_2_ and EtCO_2_. Subgroup DSEM analyses were performed to investigate relationships of physiologic variables to PbtO_2_ within patients who had either intact or impaired CO_2_ reactivity of PbtO_2_. Wilcoxon ranked sum test was used to investigate differences in median ICP, PbtO_2_, EtCO_2_, rSO_2_, ABP, PRx, and GOSE-Peds values between patients with intact and impaired CO_2_ reactivity of PbtO_2_. Statistical analyses were performed using the statistical software packages SAS 9.4 (SAS Institute, Cary, NC, USA), R Studio Version 3.4.1, and Mplus 8.1 (Muthen and Muthen 1998–2018, Los Angeles, CA, USA). 

## 3. Results

### 3.1. Patient Characteristics

Patient, trauma, clinical and neuroimaging characteristics together with neurosurgical and pharmacological interventions for each patient are summarized in Table 1. Fraction of inspired oxygen (FiO_2_) and values for hemoglobin and all of the physiologic data are summarized in Table 2. Fourteen patients were identified with severe TBI who underwent MMM with synchronized monitoring of ABP, ICP, EtCO_2_, rSO_2_, and PbtO_2_. A total of 11 patients (79%) were male. Ages ranged from 3 to 20 years (median 14.0 [IQR 11–17]). Moreover, patients (79%) were involved in a motor vehicle accident of which were automobile vs. pedestrian, 8 automobile vs. bicycle, 1 automobile vs. motorcycle, and 1 all-terrain vehicle (ATV) accident. Among the 3 patients who sustained falls, 1 experienced a crush injury from a ground-level fall, 1 experienced a blast injury after a ground level fall, and 1 patient suffered a closed head injury from a fall greater than 3 feet. GCS scores on admission ranged from 3 to 7 (median 4.0 [IQR 3.3–5.8]). GOSE-Peds scores at 12-months post-injury ranged from 1–8 (median 5.0 [IQR 3.0–5.8]). Among all, eleven patients had reactive pupils to light while one had unilaterally and two bilaterally fixed pupils on admission. Neuroimaging of the patients revealed diverse pathologic findings described in Table 1. All but 2 patients underwent neurosurgical interventions including decompressive craniectomy (8/12), hematoma evacuation (9/12) and external ventricular drainage catheter insertion (4/12). Furthermore 5 out of 14 patients (36%) underwent multimodality neurologic monitoring without a decompressive craniectomy or intracranial hematoma evacuation. All of the patients were intubated and mechanically ventilated. FiO_2_ values ranged from 30 to 95% for patients during their time of analysis. Among all, 8 patients (57%) received infusions of vasoactive agents during their analysis period, with all eight patients receiving norepinephrine infusion and two patients receiving a concurrent epinephrine or vasopressin infusion. All of the patients received sedative infusion therapies with fentanyl in 14 (100%), propofol in 8 (57%), dexmedetomidine in 4 (29%), and pentobarbital in 2 (14%). A total of 9 (64%) patients received hypertonic saline while 3 (21%) received additional mannitol infusion prior to or during their analysis period. 

### 3.2. CO_2_ Reactivity of PbtO_2_

Correlations of physiologic variables for each individual patient and overall group are presented in Table 3. We observed that at the level of grouped analysis, there was a weak positive association between PbtO_2_ and EtCO_2_ (SRC 0.05, 95% CI [0.04, 0.06]) with 9 patients demonstrating positive associations (intact CO_2_ reactivity of PbtO_2_) and 5 patients demonstrating negative associations (impaired CO_2_ reactivity of PbtO_2_). Grouped analysis demonstrated a weak positive association between PbtO_2_ and ICP (SRC 0.02, 95% CI [0.02, 0.03]) with 7 patients demonstrating positive associations and seven patients demonstrating negative associations. With respect to ABP, grouped analysis revealed a positive association with PbtO_2_, with all of the patients demonstrating such positive associations (SRC 0.36. 95% CI [0.35,0.36]. Grouped analysis demonstrated a positive association between PbtO_2_ and rSO_2_ (SRC 0.02, 95% CI [0.01, 0.02]) with 7 patients demonstrating positive associations and 7 patients demonstrating negative associations. 

Subgroup analysis of patients with intact and impaired CO_2_ reactivity of PbtO_2_ is summarized in Table 4, and differences in physiologic values between each group is summarized in Table 5. We observed a positive association between PbtO_2_ and ICP in patients with intact CO_2_ reactivity of PbtO_2_ (SRC 0.22, 95% CI [0.21, 0.23]), whereas we observed a negative association between ICP and PbtO_2_ in the impaired group (SRC −0.28, 95% CI [−0.29, −0.28]. We observed a negative association between PbtO2 and rSO_2_ in patients with intact CO_2_ reactivity of PbtO_2_ (SRC −0.08, 95% CI [−0.09, −0.08]), whereas we observed a positive association between PbtO_2_ and rSO_2_ in the impaired group (SRC 0.15, 95% CI [0.14, 0.16]. In comparison to patients with intact CO_2_ reactivity of PbtO_2_, those with impaired reactivity were observed to have decreased values of PbtO_2_, EtCO_2_ and ABP, as well as increased values of ICP, PRx, and rSO_2_. Lower GOSE-PEDs scores, reflective of improved functional outcomes, were observed in patients with intact CO_2_ reactivity of PbtO_2_ as compared to patients with impaired CO_2_ reactivity of PbtO_2_.

## 4. Discussion

In this exploratory study, we have investigated CO_2_ reactivity of PbtO_2_ by analyzing temporal relationships of PbtO_2_ and EtCO_2_ in pediatric severe TBI patients. Whereas most patients had an expected positive association between PbtO_2_ and EtCO_2_, we identified a subset of patients who had impaired CO_2_ reactivity of PbtO_2_. Patients within this subset had negative associations between ICP and PbtO_2_ in addition to higher ICP and PRx values, lower PbtO_2_ values and increased GOSE-Peds scores reflective of unfavorable outcome. These results support the notion that EtCO_2_ changes may be inversely coupled with PbtO_2_ in select pediatric TBI patients with a physiologic profile that manifests with increased BBB breakdown, impaired CVPR, increased risk of brain tissue hypoxia and increased risk of long-term functional impairments. 

A growing body of evidence supports the argument that brain tissue hypoxia is associated with unfavorable outcomes after pediatric TBI. One cohort study of 46 children with TBI observed that PbtO_2_ levels of ≥30 mmHg represented the highest sensitivity and specificity for favorable outcome [17], and a separate pediatric TBI observational cohort study of 52 children suggested that PbtO_2_ levels < 10 mmHg are associated with unfavorable outcomes [18]. This work has helped formulate the most recent level III recommendations in pediatric TBI guidelines to maintain PbtO_2_ levels > 10 mmHg in children [3]. The recent BOOST II study represented a randomized clinical trial of adult patients with TBI in which patients randomized to ICP plus PbtO_2_ monitoring had reduced time with brain tissue hypoxia and trends toward improved outcomes, as compared to patients undergoing ICP monitoring alone [19]. This has formulated the ongoing BOOST III clinical trial, which is powered to investigate whether PbtO_2_-based therapy improves outcomes in adults with TBI (ClinicalTrials.gov Identifier: NCT03754114). While evidence links low PbtO_2_ values with poor outcomes, the proposed interventions to optimize levels include raising ABP with vasopressors, optimizing hemoglobin concentration, and increasing PaCO_2_ to augment CBF [6]. Such proposed interventions arise from both adult literature and other underling etiologies (i.e., aneurysmal subarachnoid hemorrhage), making them potentially less translatable to pediatric TBI where diverse pathophysiology can arise. A more comprehensive understanding is needed in pediatric TBI patients to understand situations in which PbtO_2_ is influenced by ABP, PaCO_2_, or ICP in order to optimize its value and potentially improve outcomes. 

**Figure 1:**
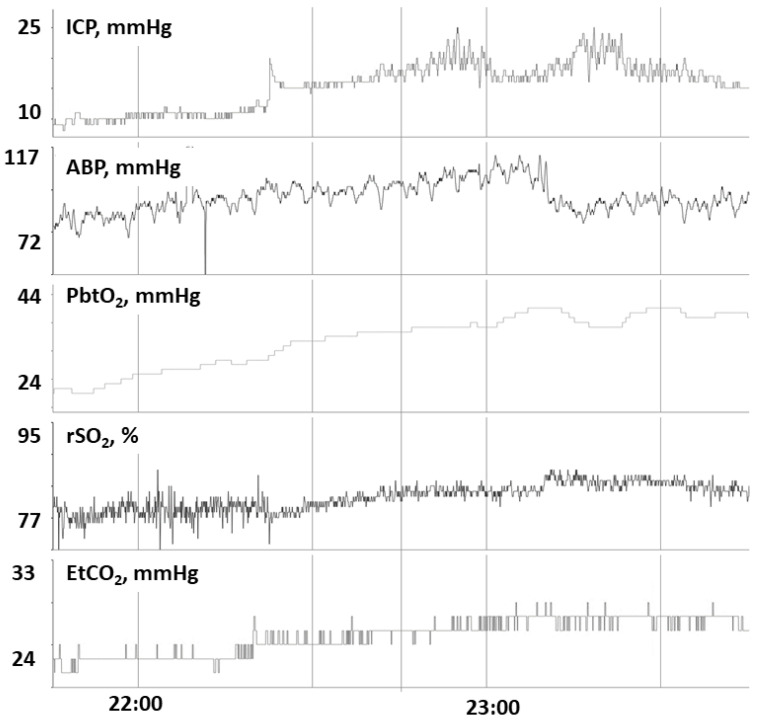
In patient 6, we observe that a rise in EtCO_2_ corresponds with a concordant rise in PbtO_2_, rSO_2_ and ICP, consistent with intact CO_2_ reactivity to PbtO_2_. Abbreviations: ICP, intracranial pressure; ABP, arterial blood pressure; PbtO_2_, partial pressure of brain tissue oxygenation; rSO_2_, cerebral regional somatic oximetry; EtO_2_, end-tidal carbon dioxide; mmHg, millimeters of mercury; %, percentage.

**Figure 2:**
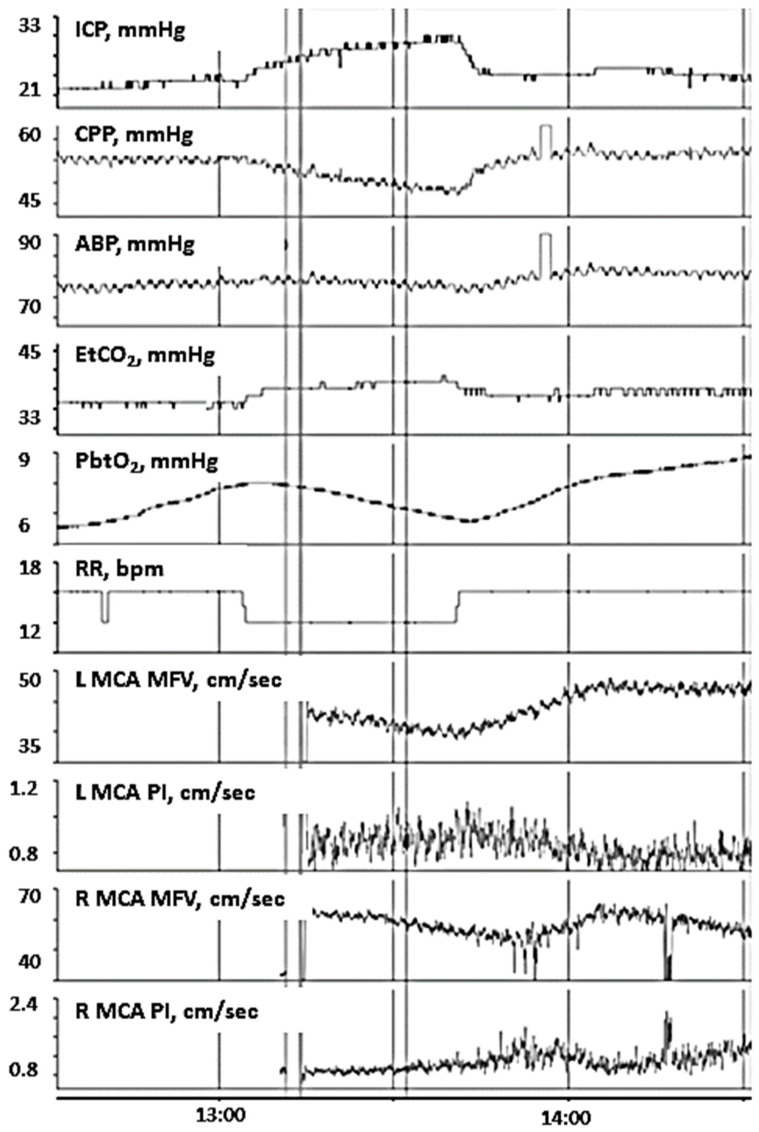
In patient 14, changes in EtO_2_ are positively associated with changes in ICP but is negatively associated with changes in PbtO_2_. This is indicative of impaired CO_2_ reactivity to PbtO_2_. Continuous transcranial Doppler ultrasound (TCD) is performed at the same time to assess changes in CBF, demonstrating changes in EtO_2_ are negatively associated with bilateral MCA MFV and positively associated with PIs. These findings suggest that increased EtO_2_ may increase cerebral edema and lead to resultant intracranial hypertension, brain tissue hypoxia, and resistive CBF in the major basal arteries. Abbreviations: ICP, intracranial pressure; CPP, cerebral perfusion pressure; ABP, arterial blood pressure; EtO_2_, end-tidal carbon dioxide; PbtO_2_, partial pressure of brain tissue oxygenation; RR, respiratory rate; bpm, breaths per minute; L, left; R, right; MCA, middle cerebral artery; MFV, mean flow velocities; PI, pulsatility index; cm, centimeters; sec, second; mmHg, millimeters of mercury.

The risk of disruption in the BBB is high after pediatric TBI. An intact BBB is essential for maintaining brain volume at a very constant level [20]. When BBB is disrupted, an increase in transcapillary hydrostatic pressure, such as through an increase in ABP or decrease in transcapillary oncotic pressure might lead toward intracranial hypertension and resultant vasogenic edema. In the injured brain, ineffective CVPR can contribute toward increased hydrostatic capillary pressure further complicating intracranial hypertension [9]. In such a microenvironment where the BBB is not intact and the CVPR is inefficient, an increase in PaCO_2_ may contribute to an increase in cerebral edema by increasing CBF and cerebral blood volume and result in a decrease in PbtO_2_ and increase in ICP. The findings we observed are supportive of this notion. Patients we observed with intact CO_2_ reactivity of PbtO_2_ had positive associations of PbtO_2_ and ICP, reflecting that increases in ICP may relate to increases in cerebral blood volume blood volume with concordant rises in PbtO_2_ (Figure 1). In contrast, patients with impaired CO_2_ reactivity of PbtO_2_ had negative associations of PbtO_2_ and ICP, which may reflect increases in cerebral edema with concomitant intracranial hypertension may reduce PbtO_2_. Concordant monitoring with continuous transcranial Doppler ultrasound in Case 14 further demonstrate such phenomena with changes in CBF (Figure 2). Furthermore, increased PRx values, reflective of decreased CVPR efficiency, were observed in the subgroup with impaired CO_2_ reactivity, also consistent with the notion that such patients have worsened cerebral edema and BBB breakdown. From these findings, we speculate that disrupted BBB and ineffective CVPR may contribute toward an inverse relationship between EtCO_2_ and PbtO_2_.

Another explanation to the inverse correlation between PbtO_2_ and EtCO_2_ might be impaired CO_2_ vasoreactivity. In a previous study, Lee et al. found that ICP > 20 mm Hg, low baseline CPP, early post-injury hypotension and hypoxia were associated with impairment of CO_2_ reactivity [21]. They showed that during the first 2 weeks after moderate and severe TBI, CO_2_ reactivity remained relatively intact but cerebral autoregulation variably was impaired. As our recordings were in the very acute stages of the injury, this explanation might not be valid for our patients.

We also observed that patients with intact CO_2_ reactivity of PbtO_2_ had negative associations between PbtO_2_ and rSO_2_, whereas patients with impaired reactivity had positive associations between each measure of cerebral oximetry. Furthermore, higher values of rSO_2_ were observed in the impaired group as compared to the intact group. These findings may reflect that patients with intact CO_2_ reactivity had increased oxygen extraction with increased metabolic demand, which may reflect better in rSO_2_ values that are more likely to reflect venous blood [22]. rSO_2_ does carry substantial technical limitations in its ability to reflect true changes in brain tissue oxygenation, and thus we adopt caution in our interpretation of these findings. 

This study was limited by single-center data collection, small sample size, and a retrospective design. Considering this, 5-h epochs were selected to minimize the impact that external factors (e.g., increasing scalp edema, fluctuating FiO_2_ levels) might have to compound PbtO_2_ and rSO_2_ values, although bias may arise from selection of those epochs. Outside of unique circumstances such as described in Figure 2, direct measures of CBF were not investigated in this study. Relationships of PbtO_2_ with other hemodynamic factors may change with respect to longer periods of time and specific medical interventions, and this requires additional investigation. While we speculate that differences in physiologic inter-relationships of PbtO_2_ and EtCO_2_ may be related to BBB breakdown, we did not investigate neuroimaging or serological biomarkers of BBB breakdown, and this would be helpful in future studies to investigate the physiologic impact of BBB integrity. Despite our attempts to minimize and remove all artifacts, there is a possibility that remaining artifacts might also cause this inverse correlation as well. We did observe lower GOSE-Peds scores at 12-months post-injury in patients with intact CO_2_ reactivity of PbtO_2_, as compared to impaired reactivity. This raises the possibility that patients with intact CO_2_ reactivity may have improved outcomes, and further work is needed in this regard. Patients with impaired CO_2_ reactivity of PbtO_2_ may benefit from alternative strategies to augment PbtO_2_ levels and improve their recovery trajectory. Our study is intended as an exploratory study for hypothesis generation, and it is not powered to assess the degree to which impaired CO_2_ reactivity of PbtO_2_ may impact secondary brain insult propagation and long-term functional outcomes. Larger prospective studies are needed in TBI patients who undergo concurrent PbtO_2_ and MMM with standardized approaches to ABP and EtCO_2_ manipulation to understand factors that influence PbtO_2_ trends, and individualized clinical management strategies that may optimize PbtO_2_ values and improve functional outcomes. 

## 5. Conclusions

After pediatric TBI, CO_2_ reactivity of PbtO_2_ can be heterogenous. Further research is needed to clarify the clinical value to which trends in EtCO_2_ monitoring can evaluate changes in cerebral oxygenation in pediatric TBI management, and to investigate individualized management strategies that can optimize PbtO_2_ levels and improve functional outcomes.

## Figures and Tables

**Table 1:** Patient Demographics.

Patient	Sex	Race	Age (Years)	TBI Cause	TBI Mechanism	TBI Type	GCS	GOSE-Peds, 12 Months Post-Injury	Radiographic Neuropathology (CT/MRI)	Neurosurgical Procedures before Monitoring	Sedation	Vasoactive Agents	Hyperosmolar Therapy	PbtO_2_ Location
1	F	Hispanic	12	MVA (Auto vs. pedestrian)	Closed	DI	4	3	Diffuse cerebral edema R convexity SDH, Mid-line shift, Cerebellar tonsillar herniation	DC and epidural hematoma evacuation	FNT (1 mcg/hg/h), PRP (50 mcg/kg/min)	None	HTS (bolus)	LF
2	M	Hispanic	3	Fall from trampoline	Crush	FFT	6	5	R SDH R to L mid-line shift. Skull base frx	Subdural hematoma evacuation	FNT (1 mcg/kg/h), Dex (0.4 mcg/kg/h)	None	None	LF
3	M	Asian	16	FL	Blast	GLF	4	1	R scalp hematoma R parietal skull frx R- frontotemporal and parietal SDH R to L mid-line shift R frontal tSAH	DC and subdural hematoma evacuation	FNT (2 mcg/kg/h), Pentobarbital (1 mg/kg/h)	NE	None	RF
4	M	Hispanic	11	MVA (Auto vs. pedestrian)	Closed	DI	4	8	L parietal scalp hematoma, EDH extending from CCJ to supraclinoid region, tSAH, diffuse cerebral edema, DAI, cerebellar edema and contusion, AO dislocation, T12-L1 frx	Bedside EVD	FNT (1 mcg/kg/h)	Antihypertensives	HTS (bolus)	LF
5	M	Native American	15	MVA (Auto vs. pedestrian)	Closed	DI	3	3	L frontotemporal and parietal skull fractures, B/L temporal contusions, B/L SDH, diffuse cerebral edema, pneumocephalus, diffuse tSAH at basal cisterns, cerebellar herniation, L to R mid-line shift	DC and epidural hematoma evacuation	FNT (1 mcg/kg/h)	NE	HTS (infusion)	RF
6	M	Caucasian	14	Fall from height	Closed	FFT	3	2	R temporal bone, occipital condyle and sphenoid sinus frx, cortical contusion on the L mid frontal region of lateral ventricles, SDH	No operation	FNT (2 mcg/kg/h)	NE	HTS (infusion)	RF
7	M	Caucasian	15	MVA (Auto vs. bicycle (w/o helmet))	Closed	DI	3	7	L-post scalp hematoma, B/L SDH, tSAH, punctate parenchymal hemorrhages, basal cistern effaced, L- Temporal frx	EVD placement at the OR	DEX (0.6 mcg/kg/h), FNT (1 mcg/kg/h), PRP (0.25 mcg/kg/min)	None	None	LF
8	M	Caucasian	14	MVA (Auto vs. motorcycle (w/o helmet))	Closed	DI	4	2	B/L EDH, SDH, effacement of basal cistern, diffuse cerebral edema	DC and epidural hematoma evacuation	DEX (1 mcg/kg/h), PRP (50 mcg/kg/min), FNT (1 mcg/kg/h)	NE	Mannitol, HTS (infusion)	RF
9	M	Hispanic	14	MVA (Auto vs. pedestrian)	Closed	DI	5	5	L post scalp hematoma, L frontal SDH, IVH, punctate parenchymal hemorrhages	N/A	FNT (1 mcg/kg/h), PRP (50 mcg/kg/min)	EPI, NE	None	LF
10	F	Caucasian	17	MVA (Auto vs. pedestrian)	Closed	DI	3	5	Scalp hematoma, posterior sutural diastases, pneumocephalus, right sigmoid sinus and superior sagittal sinus thrombus, B/L frontal lobe contusions, tSAH, SDH	DC, evacuation of R frontal contusion	FNT (1 mcg/kg/h), PRP (60 mcg/kg/min)	NE	HTS (infusion), Mannitol	LF
11	F	Native American	7	MVA (Auto vs. pedestrian)	Closed	DI	6	4	B/L frontal, parietal, temporal bone frx, open and depressed, skull base and MF frx, pneumocephalus, EDH, SDH, tSAH	DC and epidural hematoma evacuation	FNT (1 mcg/kg/h), Pentobarbital (2 mg/kg/h)	NE, Vasopressin	HTS (bolus and infusion)	RF
12	M	Hispanic	17	MVA (Auto vs. Pedestrian)	Closed	DI	5	5	Central midbrain hemorrhage, B/L IVH, R temporal lobe ICH, tSAH, SDH	EVD placement	DEX (0.2 mcg/kg/h), FNT (3 mcg/kg/h), PRP (25 mcg/kg/min)	None	None	RF
13	M	Hispanic	20	MVA (ATV head on head crush (w/o helmet))	Closed	DI	7	6	R- frontotemporal scalp hematoma, depressed frx and contusion, R-FPT and occipital ICH, IVH, diffuse cerebral edema, R-to-L midline shift	DC and intraparenchymal hematoma evacuation, partial frontal lobectomy, EVD placement	FNT (3 mcg/kg/h), PRP (25 mcg/kg/min)	None	Mannitol (bolus) HTS (bolus)	RF
14	M	Caucasian	14	MVA (Auto vs. pedestrian)	Closed	DI	7	6	R parietal scalp hematoma, R-TFP ICH, retroclinoid extradural hematoma, tSAH	DC and hematoma evacuation	FNT (1 mcg/kg/h), PRP (30 mcg/kg/min)	NE	HTS (bolus)	RF

Abbreviations: TBI, traumatic brain injury; GCS, Glasgow Coma Scale; GOSE-Peds, Glasgow Outcome Scale—Extended Pediatrics; CT, computed tomography; MRI, magnetic resonance imaging; Auto, automobile; FL, fall; w/o, without; DI, diffuse impact; FFT, fall from greater than 3 feet; GLF, ground level fall; R, right; SDH, subdural hematoma; L, left; tSAH, traumatic subarachnoid hemorrhage; B/L, bilateral; DC, frx, fracture; CCJ, cervicocranial junction; DAI, diffuse axonal injury; AO, atlanto-occipital; IVH, intraventricular hemorrhage; decompressive craniectomy; EVD, external ventricular drain; FNT, Fentanyl; DEX, Dexmetomidine; EPI, epinephrine; PRP, propofol; NE, norepinephrine; HTS, hypertonic saline; mcg, micrograms; kg, kilograms; min, minute; h, hour; LF, left frontal; RF, right frontal.

**Table 2:** Patient PbtO_2_ Location, Hemoglobin Concentration, and Physiologic Values.

Patient	PbtO_2_ Location	Hemoglobin Concentration (g/dL)	FiO_2_, sta%	Median PbtO_2_, mmHg	Median EtCO_2_, mmHg	Median ABP, mmHg	Median ICP, mmHg	Median rSO_2_, %	Median PRx
1	LF	10.9	70–90	10.0 [9.0, 13.0]	38.0 [33.0, 41.0]	79.0 [77.0, 81.6]	14.0 [11.0, 14.0]	95.0 [93.0, 95.0]	−0.06 [−0.34, 0.12]
2	LF	10.6	40–50	69.0 [65.0, 73.0]	32.0 [31.0, 32.0]	93.0 [91.0, 97.0]	20.0 [17.0, 21.0]	76.1 [74.2, 77.0]	0.18 [−0.51, 0.28]
3	RF	11.1	55–60	21.0 [19.0, 24.0]	33.0 [32.0, 35.0]	87.0 [83.0, 90.0]	11.0 [10.0, 15.0]	77.0 [76.0, 84.4]	−0.40 [−0.73, −0.22]
4	LF	13.5	50	54.0 [47.0, 58.0]	30.0 [29.0, 32.0]	92.0 [90.0, 94.0]	7.0 [7.0, 8.0]	79.0 [78.0, 80.0]	0.19 [−0.03, 0.47]
5	RF	7.5	5–50	52.0 [50.0, 64.0]	38.0 [37.0, 40.0]	76.8 [73.1, 79.0]	6.0 [5.0, 7.0]	75.0 [71.5, 76.7]	0.29 [0.06, 0.51]
6	RF	10.2	35–80	39.0 [37.0, 44.0]	32.0 [31.0, 36.0]	109.0 [100.0, 111.0]	11.0 [10.0, 12.0]	71.4 [70.1, 73.0]	0.25 [0.02, 0.48]
7	LF	11.4	45–60	27.0 [25.0, 28.0]	33.0 [32.0, 34.0]	91.0 [84.0, 98.0]	6.0 [5.0, 8.0]	69.0 [64.8, 71.0]	−0.11 [−0.30, 0.07]
8	RF	11.6	50–65	7.0 [5.0, 10.0]	37.0 [35.0, 39.0]	78.0 [75.0, 82.0]	15.0 [14.0, 16.0]	91.0 [89.0, 92.0]	0.07 [−0.21, 0.35]
9	LF	6.2	40–60	21.1 [14.5, 27.1]	28.0 [27.0, 29.0]	76.0 [73.0, 78.0]	7.0 [7.0, 10.0]	82.0 [81.0, 84.0]	0.67 [0.54, 0.82]
10	LF	9.0	30–95	14.2 [12.6, 18.6]	32.0 [31.0, 33.0]	81.0 [76.0, 83.5]	12.0 [10.0, 13.0]	95.0 [94.0, 95.0]	−0.06 [−0.30, 0.18]
11	RF	9.8	60–85	26.0 [25.0, 28.0]	33.0 [32.0, 34.0]	100.0 [960, 103.0]	11.0 [7.0, 14.0]	33.0 [70.0, 75.4]	0.07 [−0.29, 0.40]
12	LF	11	40–50	76.6 [74.9, 78.9]	36.0 [34.0, 39.0]	75.0 [68.0, 80.0]	16.0 [9.0, 19.0]	71.8 [70.0, 73.1]	−0.02 [−0.22, 0.20]
13	RF	15.9	30–100	36.0 [32.0, 38.0]	33.0 [33.0, 34.0]	93.0 [91.0, 99.0]	14.0 [12.1, 15.0]	74.8 [73.0, 75.9]	0.15 [−0.01, 0.29]
14	RF	16.2	40–50	8.5 [6.9, 9.1]	37.0 [33.0, 38.0]	81.0 [78.0, 87.7]	23.0 [22.0, 24.0]	95.0 [95.0, 95.0]	0.13 [−0.05, 0.31]

Median data is presented with both the median value as well as the interquartile range in brackets. Abbreviations: PbtO_2_, brain tissue oxygenation; g, gram; dL, deciliter; FiO_2_, fraction of inspired oxygen; mmHg, millimeters of mercury; LF, left frontal; RF, right frontal, %, percentage; IQR, interquartile range.

**Table 3:** Physiologic Relationships of PbtO_2_ to ICP, ABP and rSO_2_.

Patient	PbtO_2_ to EtCO_2_ [SRC (95% CI)]	PbtO_2_ to ICP [SRC (95% CI)]	PbtO_2_ to ABP [SRC (95% CI)]	PbtO_2_ to rSO_2_ [SRC (95% CI)]
1	0.06 (0.05, 0.08)	0.30 (0.28, 0.31)	0.06 (0.05, 0.08)	0.24 (0.22, 0.25)
2	−0.06 (−0.07, −0.05)	0.04 (0.03, 0.06)	0.14 (0.13, 0.15)	0.24 (0.22, 0.25)
3	0.20 (0.19, 0.22)	−0.04 (−0.05, −0.02)	0.23 (0.21, 0.24)	−0.35 (−0.36, −0.33)
4	0.26 (0.24, 0.27)	0.14 (0.12, 0.15)	0.48 (0.47, 0.49)	−0.24 (−0.25, −0.22)
5	0.18 (0.17, 0.20)	0.49 (0.48, 0.50)	0.21, (0.20, 0.22)	0.37 (0.36, 0.38)
6	0.83 (0.82, 0.83)	0.57 (0.56, 0.59)	0.53 (0.52, 0.55)	0.39 (0.37, 0.41)
7	0.64 (0.63, 0.64)	−0.54 (−0.55, −0.53)	0.38 (0.37, 0.39)	−0.44 (−0.45, −0.42)
8	0.14 (0.12, 0.15)	0.14 (0.13, 0.16)	0.63 (0.62, 0.63)	−0.33 (−0.34, −0.32)
9	−0.44 (−0.45, −0.42)	−0.25 (−0.26, −0.24)	0.66 (0.65, 0.67)	0.20 (0.19, 0.21)
10	−0.45 (−0.46, −0.43)	−0.40 (−0.41, −0.39)	0.46 (0.45, 0.47)	0.26 (0.25, 0.28)
11	−0.65 (−0.66, −0.65)	−0.58 (−0.59, −0.57)	0.03 (0.02, 0.05)	−0.48 (−0.49, −0.47)
12	0.09 (0.08, 0.11)	−0.03 (−0.04, −0.01)	0.42 (0.41, 0.43)	0.08 (0.06, 0.09)
13	0.59 (0.58, 0.60)	0.16 (0.15, 0.18)	0.67 (0.67, 0.68)	−0.21 (−0.23, −0.20)
14	−0.45 (−0.47, −0.44)	−0.23 (−0.24, 0.22)	0.48 (0.46, 0.49)	−0.12 (−0.14, −0.11)
Grouped Analysis	0.05 (0.04, 0.06)	0.02 (0.02, 0.03)	0.36 (0.35, 0.36)	0.02 (0.01, 0.02)

Abbreviations: PbtO_2_, partial pressure of brain tissue oxygenation; EtCO_2_, end-tidal carbon dioxide; ICP, intracranial pressure; ABP, arterial blood pressure; rSO_2_, cerebral regional somatic oximetry; SRC, standardized regression coefficient; CI, credible interval.

**Table 4:** Subgroup Analysis of Patients with Intact and Impaired CO_2_ Reactivity of PbtO_2_.

CO_2_ Reactivity of PbtO_2_	PbtO_2_ to EtCO_2_ [SRC (95% CI)]	PbtO_2_ to rSO_2_ [SRC (95% CI)]	PbtO_2_ to ICP [SRC (95% CI)]	PbtO_2_ to ABP [SRC (95% CI)]
Intact	0.44 (0.44, 0.45)	−0.08 (−0.09, −0.08)	0.22 (0.21, 0.23)	0.38 (0.38, 0.39)
Impaired	−0.38 (−0.39, −0.37)	0.15 (0.14, 0.16)	−0.28 (−0.29, −0.28)	0.31 (0.31, 0.32)

Abbreviations: CO_2_, carbon doixide; PbtO_2_, partial pressure of brain tissue oxygenation; EtCO_2_, end-tidal carbon dioxide; ICP, intracranial pressure; ABP, arterial blood pressure; rSO_2_, cerebral regional somatic oximetry; SRC, standardized regression coefficient; CI, credible interval.

**Table 5:** Physiologic and Outcome Differences Between Patients with Intact and Impaired CO_2_ Reactivity of PbtO_2_.

CO_2_ Reactivity to PbtO_2_	Intact CO_2_ Reactivity of PbtO_2_, Median [IQR]	Impaired CO_2_ Reactivity of PbtO_2_, Median [IQR]	*p*-Value
PbtO_2_	36.0 [21.0, 52.0]	21.1 [14.2, 26.0]	0.0000
ICP	11.0 [7.0, 14.0]	12.0 [11.0, 20.0]	0.0000
PRx	0.07 [−0.06, 0.19]	0.13 [0.07, 0.18]	0.0134
ABP	87.0 [78.0, 92.0]	81.0 [81.0, 93.0]	0.0000
EtCO_2_	33.0 [33.0, 37.0]	32.0 [32.0, 33.0]	0.0000
rSO_2_	75.0 [71.8, 79.0]	82.0 [76.1, 95.0]	0.0000
GOSE-Peds, 12 months post-injury	3.0 [2.0, 6.0]	5.0 [5.0, 5.0]	0.0000

Median data is presented with both the median value as well as the interquartile range in brackets. Abbreviations: CO_2_, carbon dioxide; PbtO_2_, partial pressure of brain tissue oxygenation; EtCO_2_, end-tidal carbon dioxide; ICP, intracranial pressure; ABP, arterial blood pressure; rSO_2_, cerebral regional somatic oximetry; PRx, pressure reactivity index; IQR, interquartile range.

## Data Availability

The raw data supporting the conclusions of this article can be made available by the corresponding author, without undue reservation.
